# Scientific Rationale for a Bottom-Up Approach to Target the Host Response in Order to Try and Reduce the Numbers Presenting With Adult Respiratory Distress Syndrome Associated With COVID-19. Is There a Role for Statins and COX-2 Inhibitors in the Prevention and Early Treatment of the Disease?

**DOI:** 10.3389/fimmu.2020.02167

**Published:** 2020-09-02

**Authors:** Geoffrey Mark Verrall

**Affiliations:** ^1^South Australian Sports Institute, Adelaide, SA, Australia; ^2^Sports and Arthritis Clinic, Adelaide, SA, Australia

**Keywords:** COVID-19, COX-2 inhibitors, statins, immunomodulatory, Diclofenac

## Abstract

The inflammatory response to and the subsequent development of Adult Respiratory Distress Syndrome (ARDS) is considered to underpin COVID-19 pathogenesis. With a developing world catastrophe, we need to examine our known therapeutic stocks, to assess suitability for prevention and/or treatment of this pro-inflammatory virus. Analyzing commonly available and inexpensive immunomodulatory and anti-inflammatory medications to assess their possible effectiveness in improving the host response to COVID-19, this paper recommends the following: (1) optimize current health—cease (reduce) smoking, ensure adequate hypertension and diabetes control, continue exercising; (2) start on an HMG CoA reductase inhibitor “statin” for its immunomodulatory and anti-inflammatory properties, which may reduce the mortality associated with ARDS; and (3) consider using Diclofenac (or other COX-2 inhibition medications) for its anti-inflammatory and virus toxicity properties. For purposes of effectiveness, this needs to be in the early course of the disease (post infection and/or symptom presentation) and given in a high dose. The downsides to these recommended interventions are considered manageable at this stage of the pandemic.

## Introduction

With an emergency response needed for an emerging viral disease, such as COVID-19, it is unlikely a top-down approach to the development of specific vaccine and drugs will be possible or even effective in the time frame required to meet such a threat. What can be examined is a bottom-up approach, hopefully with some common easily obtainable and inexpensive medications, that can target the host response to the virus (1).

This manuscript involves understanding the mechanism of pathophysiology of disease and previous experiments (mostly laboratory based) to understand how a treatment works and empirical direct observations that the proposed treatments do work.

## The Pathogenesis of SARS-CoV 2 (COVID-19)

Much of our information about COVID-19 comes from the more lethal but less communicable SARS-CoV epidemic. The considered pathogenesis is an inflammatory response of the lung cells that overwhelms the system with a cytokine storm (2). Cytokines are proteins that orchestrate inflammatory response. Risk factors include being older and having hypertension and diabetes. However, the epidemiology and risk factors are not entirely clear with this newly recognized virus.

The entry point for the virus is the Angiotensin Converting Enzyme 2 (ACE2) receptor located on epithelial cells (3). ACE2 receptors are proteins on the surface of many cell types with a high abundance on the Type 2 pneumocytes of the lungs. The spike-like protein of the SARCoV2 binds to ACE2 allowing entry and infection of cells. A host-mediated response then occurs with an induction of the inflammatory (Cyclo-Oxygenase Enzyme-2) COX-2 enzyme (2). This results in a pro-cytokine (inflammatory) cascade (2). In some hosts, this leads to Adult Respiratory Distress Syndrome (ARDS), which has a high lethality. The general time from symptom presentation to the subsequent development to ARDS is approximately 4–10 days with a progressive worsening of the condition during that time. It is not yet understood why some people when infected with COVID-19 get a relatively mild illness whereas others respond with an inflammatory response that can result in ARDS.

The COX-2 enzyme is not expressed in many tissues, including the lung, except when associated with inflammation. The role of the COX-2 enzyme has a poorly understood role in immunity (4). Viral infection COX-2 enzyme induction occurs in a complex process with the consequential immunity response being associated with the production of cytokines, inflammatory prostanoids, and increased vascular permeability (4).

## Aims of Treatment

Any treatment using the bottom-up approach is aimed at improving the immunity of the entire host. Immunomodulation would then need to be protective, enhancing host immune health, and have treatment, anti-cytokine storm (anti-inflammatory) properties. It is likely that during bout of severe infection, different elements of the immune system will need enhancing, whereas other parts need suppressing. It is also likely that this may change during the infection, with unknown and unpredictable timeframes associated with this required alteration. Obviously, you need the “right” immunomodulating drug at the “right” time as administration at the wrong time could worsen clinical outcome. With current knowledge of any condition, and particularly a new emerging threat such as COVID-19, this is a difficult task.

In broad terms ARDS, the condition responsible for the mortality associated with COVID-19 may be classified into hyper- and hypo-inflammatory subphenotypes. With emerging virus infections these are generally considered to be hyper-inflammatory. Immediate cessation of smoking along with better hypertension/diabetes control would immediately assist in improving the bodies hyper-inflammation.

Other host responses that can be targeted with the specific need of trying to assist in decreasing ARDS presentations associated with the current COVID-19 pandemic include altering the lung endothelial cell membrane to decrease virus incorporation, decreasing the cytokine response with virus presentation to the endothelial cell membrane, altering the amount of lung host cell numbers to try and prevent virus incorporation, inhibiting the direct cell-to-cell spread (a common viral pathogenic mechanism), improving viral toxicity to prevent viral replication, and inhibiting inflammatory pathways after virus infection.

## Endothelial Cell Membrane and Hmgcoa Reductase Inhibitors (Statins)

It has been demonstrated that there is a 30% reduction in mortality on those patients admitting to hospital with pneumonia if they were taking HMGCoA reductase inhibitors “statins” prior to admission (5). With the study being performed retrospectively, it was also concluded that those patients taking both statins with a combination of either ACEI (Angiotensin Converting Enzyme Inhibitors) or an ARB (Angiotensin receptor blockers) experience even less lethality (6). Having both drugs in combination provided a more synergistic effect (6). Detailed information on the dose of statins was not disclosed, but there was slightly more protection from mortality when taking a higher dose of the statin simvastatin (5). The study was performed in the first decade of this century when simvastatin was more commonly prescribed than other statins, such as atorvastatin and rosuvastatin, and making a definitive conclusion about the effectiveness of one statin over another is thus not possible. Additionally, there was no statistical comparison performed between the different doses of statins.

In a randomized controlled trial (7) on patients diagnosed with ARDS there was no clinical benefit, morbidity or mortality, in those given Simvastatin compared to placebo. However, when the hyper-inflammatory sub phenotype of ARDS was separately analyzed there was improved survival with simvastatin compared to placebo, even when given late in the course of the disease (8). There have been more recent studies repeating this using the currently most commonly prescribed statins, Atorvastatin and rosuvastatin, with disappointing results in the use of Statins as a late treatment for ARDS (9).

Both statins and ACEI/ARB drugs are known to be anti-inflammatory and immunomodulatory. When the lung endothelial cell barrier is breached by the virus this triggers a release of Angiopoietin (Angpt-2), which in turn increases the release of pro-inflammatory cytokines (1). Statins affect the Angpt/TIE2 axis and decrease Angpt-2 whereas the ARB’s are direct angpt-2 antagonists (1). Statins have been demonstrated to inhibit airway inflammation, possibly by a pathway of attenuating RANTES release.

RANTES (regulated on activation, normal T-cell expressed and secreted) is now recognized to stimulate the influx of numerous inflammatory cells, including monocytes, eosinophils, and neutrophils. Additionally, statins attenuate viral dsRNA-induced AKT phosphorylation, which reduces viral replication.

## The Use of Statins

With respect to side-effect profile there is probably only limited downside in commencing statin drugs, considering the state of the current epidemic. However, if statins are to be used as a bottom-up approach, they need to be commenced with as much time prior to the viral infection as possible if this is to alter any underlying prevalent lung inflammation. The principal side-effects of commencing statins is associated with musculoskeletal side-effects, and these should be monitored in anyone commencing these drugs.

This recommendation for the use of Statins for treatment of SARS-CoV2 is difficult, as there are limited studies demonstrating clinical benefit once you are infected with the virus. The anti-inflammatory and immunomodulatory properties of statins need to be established prior to the host being infected.

## Alteration in the Amount of Host Cell Numbers to Influence the Disease

The ACE2 receptor on epithelial cells is the entry point for the SARS-CoV2 virus. The understanding of ACE2 has altered over recent years with it not being understood that overexpression results in better anti-hypertensive control. It is also known that ACE2 down regulates pro-inflammatory cytokines (2). In contrast, ACE2 deficiency will increase IL-6 and other similar pro-inflammatory proteins (10). There is a current argument about the role of ARB/ACEI drugs with COVID-19 that is beyond the scope of this analysis.

## Inhibition of the Inflammatory Pathways to Prevent ARDS

Viral infection activates a COX-2 inflammatory cascade that is most marked in the initial inflammatory phase (11).

Experiments demonstrated that administering a COX-2 inhibitor early in a disease course may enhance endogenous interferon, a protein that coordinates cellular anti-viral response (4). It was proposed that COX-2 inhibition could be an effective anti-viral therapy in humans to boost the anti-viral response provided it was given soon after the initiation of the infection (4). Mice with COX-2 enzyme deletion exhibited a reduced mortality to influenza (12).

With the respiratory distress associated with the H5N1 virus, the proinflammatory cascade was rapider and broader than those arising from other viral infections (13). As selective COX-2 inhibitors suppress hyper-induction of cytokines in the proinflammatory cascade, it was proposed that this knowledge could provide a basis for the possible development of novel therapeutic interventions for the treatment of hyper-inflammatory ARDS, as adjuncts to antiviral drugs (13).

Which COX-2 inhibitor should we trial for use? The rest of this analysis is devoted to that question.

## Properties of NSAIDS and Corticosteroids

All Non-Steroidal Anti-Inflammatory Drug (NSAIDs) exert their principal anti-inflammatory effect through their COX-2 inhibition. Of the commonly available NSAIDs, Diclofenac has as much COX-2 inhibition as Celecoxib and is more selective than Meloxicam (14). However, when the commonly prescribed doses are taken into account, Diclofenac 50 mg tds, with its shorter half-life (2 h), will result in more COX-2 inhibition when compared to the other COX-2 inhibitors like Refecoxib (highly selective Cox2 inhibitor) or Meloxicam (moderately selective Cox2 inhibitor) (15).

Commonly available NSAIDs that are selective COX-2 inhibitors have significantly longer half-lives (5–20 h) when compared to Diclofenac. Even Celecoxib, with the shortest half-life of 5 h of the selective COX-2 inhibitors, still needs a loading dose due its slow absorption with a plasma level peak at about day 3–4 (16). Diclofenac can reach therapeutic plasma levels far more rapidly due to its better absorption and shorter half-life. Additionally, acidic NSAIDs with high degree of protein binding (Diclofenac, Ibuprofen) more selectively accumulate and persist at sites of inflammation. This compares to the specific COX-2 inhibitors, which are non-acidic and are diffused homogenously throughout body. Diclofenac has been demonstrated to persist at the site of inflammation, as it is bound to COX-2, despite plasma clearance and kidney excretion (17).

When understanding inflammation, IL-10 (Interleukin-10) is immunosuppressive and downregulates IL-6 and the other proinflammatory cytokines. IL-6 mediates release of acute-phase proteins, which with SARS-CoV2 infection we consider as not desirable. In a randomized-controlled trial of post-surgical patients, those treated with Diclofenac, and compared to those not receiving a Diclofenac dosage, there was a decrease in IL-6, an increase in IL-10, and a smaller increase in CRP (18). It was observed that the IL-10 increased prior to the noted decrease in IL-6 and prior to the subsequent stress-induced cortisol action. The conclusion was that although prolonged high IL-10 has been associated with developing sepsis altering the immediate IL6-IL10 balance may be beneficial on reducing the acute inflammatory response (19). Other COX-2 inhibitors have been demonstrated to decrease IL-10, but these take a longer period with upregulation occurring by day 6–7 (19). In the case of specific COX-2 inhibitors, this upregulation was considered a consequence of the normal homeostasis feedback loop rather than direct induction as proposed for the drug Diclofenac (20).

In conclusion, in looking for a COX 2 inhibitor to use as an anti-inflammatory agent in early viral disease, Diclofenac is probably the best due to its short half-life, rapid mechanism of action, and superior inhibition of COX-2 at therapeutic doses.

With the early response to the COVID-19 epidemic there has been some non-scientific reports of avoiding Ibuprofen—a less selective COX-2 inhibitor (15). It is known that taking COX-1 inhibitors can prime a subsequent inflammatory cascade, which is a property of most NSAIDs. Avoiding taking these medications prior to infection is therefore recommended. It is not known whether taking COX-2 inhibitors taken prior to infection would attenuate the COX-2 inhibition when it is required.

## The Anti-Inflammatory and Anti-Viral Properties of Diclofenac Make It Suitable for Use as Treatment in the Early Stage of COVID-19 Infection

With infection there is a need for the endothelial cell endosomes to acidify in order to allow viral entry to the cell. Elevating endosomal pH by inhibiting cathepsin L, an important lysosomal endopeptidase enzyme, is the aim of another compound that has significant interest in the treatment of COVID-19 infections, hydroxychloroquine. NSAIDs, like Diclofenac, also inhibit Cathepsins L activity (21). In contrast, steroidal anti-inflammatories are not considered to inhibit this pathway (22).

It has been demonstrated that COX inhibition prevents Cytomegalovirus (CMV) from spreading the virus from cell to cell by a mechanism where the maturation and cell movement is blocked (23). To achieve this effect, high doses were needed (23). In this study another NSAID with a short half-life, Indomethacin, was used. Diclofenac, when compared to Indomethacin, also has a short half-life with similar COX-1-inhibition properties but superior COX-2-inhibition properties.

It had been considered that NSAID Cyclopentenone cyclooxygenase metabolites act against RNA viruses. COVID-19 is an RNA virus. To prove this, dogs known to have coronavirus infections were given Indomethacin and infected with SARS-CoV. When analyzed, there was a 1000-fold reduction in virus yield with the mechanism of effect being considered a blocking of viral RNA synthesis (24). However, this mechanism does not prime the host cell to raise a defense response against the virus but rather was useful when the virus had already entered the cell (24). Finally, the viral cytotoxic effect was seen at higher doses than would be needed for COX inhibition. Additionally, aspirin did not work in a similar manner (24).

Even though it is an old drug (1976) Diclofenac still has some unknown, somewhat novel, mechanisms of action which, when analyzed, may also assist in anti-viral (anti-COVID-19) action (25). Diclofenac blocks acid-sensing ion channels (25). The ion channel activity of SARS-CoV 3a protein is essential for activation of the pro-inflammatory NLRP3 inflammasome (26). Diclofenac also inhibits PPAR-γ (25). Notably, alveolar macrophages (AM) in the lungs constitutively express high levels of PPAR-γ to rapidly produce inflammatory cytokines following microbial challenge. The downregulation of PPAR-γ in the AM cells may result in beneficial functions under certain conditions (27), such as with the viral challenge to the lungs from SARS-CoV2. Diclofenac also inhibits Phospholipase-A2 (25) with binding by Diclofenac being demonstrated (28). Phospholipase-A2 is important in the pro-inflammatory response process. When comparing different drugs, Diclofenac reduced phospholipase-A2 activity by 93%, ketoprofen 90%, dexamethasone 62%, and methylprednisolone by 50% with weak inhibition of phospholipase-A2 activity being demonstrated by betamethasone and hydrocortisone (29). Finally, patients who died following SARS-CoV infection had elevated Phospholipase-A2 G2D (PLA2G2D) and were by the most part older (30). It is known that Phospholipase A2 increases in older people. Having these elevated PLA2G2D increases the level of immunosuppressive lipid mediators presumably to dampen the response to environmental antigens. It was considered that this adversely effected the protective innate response when this was required (30).

## The Use of Diclofenac (or Other COX-2 Inhibitors) in the Early Stage of COVID-19 Infection

Diclofenac is inexpensive and available throughout the world, often without medical supervision. Although relatively safe, caution needs to be observed if using such a method of treatment on a population basis. Allergy to aspirin is a contra-indication with exacerbation of asthma and gastric mucosal lining damage being common side-effects. Caution needs to be exhibited in those people with impaired renal function. However, when compared to the current morbidity and mortality of the COVID-19 pandemic, amplified with the development of ARDS, and without effective other treatments at this stage it would be considered the downside risks could be managed.

## Conclusion

This study used an analysis of the literature with a bottom-up approach to try and improve the host response to the looming (current) pandemic caused by the virus COVID-19. Following this analysis, we recommend the following: (1) optimize current health—cease (reduce) smoking, ensure adequate hypertension and diabetes control, and continue exercising; (2) with appropriate research being undertaken, commence an HMG CoA reductase inhibitor “statin” in a medium dose for its immunomodulatory and anti-inflammatory properties; and (3) consider using Diclofenac (or other COX-2 inhibition medications) for its anti-inflammatory and virus toxicity properties. For effectiveness and decreased risk, this needs to be in the early course of the disease (post infection and/or symptom presentation) and given in a high dose.

## Author Contributions

The author confirms being the sole contributor of this work and has approved it for publication.

## Conflict of Interest

The author declares that the research was conducted in the absence of any commercial or financial relationships that could be construed as a potential conflict of interest.

## References

[1] Fedson D. Treating the host response to emerging virus diseases: lessons learned from sepsis, pneumonia, influenza and Ebola. *Ann Transl Med.* (2016) 4:421. 10.21037/atm.2016.11.03 27942512 PMC5124618

[2] Cheng V Lau S Woo P Yuen K. Severe acute respiratory syndrome Coronavirus as an agent of emerging and reemerging infection. *Clin Micro Rev.* (2007) 20:660–94. 10.1128/CMR.00023-07 17934078 PMC2176051

[3] Li W Moore M Vasilieva N. Angiotensin-converting enzyme 2 is a functional receptor for the SARS coronavirus. *Nature.* (2003) 426:450–4. 10.1038/nature02145 14647384 PMC7095016

[4] Kirkby N Ziass A Wright W Jiao J Chan MV Warner TD Differential COX-2 induction by viral and bacterial PAMPs: consequences for cytokine and interferon responses and implications for anti-viral COX-2 directed therapies. *Biochem Biophys Res Comm.* (2013) 438:249–56. 10.1016/j.bbrc.2013.07.006 23850620 PMC3759847

[5] Mortensen E Nakashima B Cornell E Copeland LA Pugh MJ Anzueto A Population based study of statins, AT 2 receptor blockers (ARB’s) and ACE inhibitors on pneumonia-related outcomes. *Clin Infect Dis.* (2012) 55:1466–73. 10.1093/cid/cis733 22918991 PMC3491858

[6] Mortensen E Anzueto A. Prior use of both a statin and ARB is associated with lower mortality for patients hospitalized with pneumonia. *Eur Respir J.* (2016) 48:OA33292016 10.1183/13993003.congress-2016.OA3329

[7] McAuley DF Laffey JG O’Kane CM Perkins GD Mullan B Trinder TJ Simvastatin in the acute respiratory distress syndrome. *N Engl J Med.* (2014) 371:1695–703. 10.1056/NEJMoa1403285 25268516

[8] Calfee CS Delucchi KL Sinha P Matthay MA Hackett J Shankar-Hari M Acute respiratory distress syndrome subphenotypes and differential response to simvastatin: secondary analysis of a randomised controlled trial. *Lancet Respir Med.* (2018) 6:691–8. 30078618 10.1016/S2213-2600(18)30177-2 PMC6201750

[9] Sinha P Delucchi KL Thompson BT McAuley DF Matthay MA Calfee CS Latent class analysis of ARDS subphenotypes: a secondary analysis of the statins for acutely injured lungs from sepsis (SAILS) study. *Intensive Care Med.* (2018) 44:1859–69. 10.1007/s00134-018-5378-3 30291376 PMC6317524

[10] Kazemi-Bajestani S Patel V Wang W Oudit G. Targeting the ACE2 and apelin pathways are novel therapies for heart failure: opportunities and challenges. *Cardiol Res Prac.* (2012) 2012:823193. 10.1155/2012/823193 22655211 PMC3359660

[11] Ricciotti E FitzGerald GA. Prostaglandins and inflammation. *Arterioscler Thromb Vasc Biol.* (2011) 31:986–1000. 10.1161/ATVBAHA.110.207449 21508345 PMC3081099

[12] Carey M Bradbury J Seubert R Langenbach R Zeldin DC Germolec DR Contrasting effects of COX-1 and COX-2 deficiency on the host response to influenza A viral infection. *J Immunol.* (2005) 175:6878–84. 10.4049/jimmunol.175.10.6878 16272346

[13] Lee MY Cheung CY Peiris JS. Role of cyclooxygenase-2 in H5N1 viral pathogenesis and the potential use of its inhibitors. *Hong Kong Med J.* (2013) 19(Suppl. 4):29–35. 23775184

[14] Altman R Bosch B Brune K Patrignani P Young C. Advances in NSAID development: evolution of diclofenac products using pharmaceutical technology. *Drugs.* (2015) 75:859–77. 10.1007/s40265-015-0392-z 25963327 PMC4445819

[15] Van Hecken A Schwartz J Depre M De Lepeleire I Dallob A Tanaka W Comparative inhibitory activity of refecoxib, meloxicam, diclofenac, ibuprofen and naproxen on COX-2 versus COX-1 in healthy volunteers. *J Clin Pharmacol.* (2000) 40:1109–20. 11028250

[16] Brune K Patrignani P. New insights into the use of currently available non-steroidal anti-inflammatory drugs. *J Pain Res.* (2015) 8:105–18. 10.2147/JPR.S75160 25759598 PMC4346004

[17] Mahdy A Galley H Abdel-Wahed A el-Korny KF Sheta SA Webster NR. Differential modulation of interleukin-6 and interleukin-10 by diclofenac in patients undergoing major surgery. *Br J Anae.* (2002) 88:797–802. 10.1093/bja/88.6.797 12173196

[18] Stolina M Sharma S Lin Y Dohadwala M Gardner B Luo J specific inhibition of cyclooxygenase 2 restores antitumor reactivity by altering the balance of IL-10 and IL-12 synthesis. *J Immunol.* (2000) 164:361–70. 10.4049/jimmunol.164.1.361 10605031

[19] Sriramula A Xia H Xu P Lazartigues E. Brain-targeted ACE2 overexpression attenuates neurogenic hypertension by inhibiting COX mediated inflammation. *Hypertension.* (2015) 65:577–86. 10.1161/HYPERTENSIONAHA.114.04691 25489058 PMC4326547

[20] Blanco F Guitian R Moreno J de Toro FJ Galdo F. Effect of anti-inflammatory drugs on the COX-1 and COX-2 activity in human articular chondrocytes. *J Rhuematol.* (1999) 26:1366–73. 10381057

[21] Raghav N Kamboj RC Singh H. Effect of some steroidal & non-steroidal anti-inflammatory drugs on purified goat brain cathepsin L. *Indian J Med Res.* (1993) 98:188–92. 8262580

[22] Zielińska K Van Moortel L Opdenakker G De Bosscher K Van den Steen PE. Endothelial response to glucocorticoids in inflammatory diseases. *Front Immunol.* (2016) 7:592. 10.3389/fimmu.2016.00592 28018358 PMC5155119

[23] Schroer J Shenk T. Inhibition of cyclooxygenase activity blocks cell-to-cell spread of human cytomegalovirus. *Proc Natl Acad Sci USA.* (2008) 105:19468–73. 10.1073/pnas.0810740105 19033472 PMC2614784

[24] Amici C Di Caro A Ciucci A Chiappa L Castilletti C Martella V Indomethacin has a potent antiviral activity against SARS Coronavirus. *Antivir Ther.* (2006) 22:1021–30. 17302372

[25] Gan TJ. Diclofenac: an update on its mechanism of action and safety profile. *Curr Med Res Opin.* (2010) 26:1715–31. 10.1185/03007995.2010.486301 20470236

[26] Chen I Moriyama M Chang M Ichinohe T. Severe acute respiratory syndrome Coronavirus viroporin 3a activates the NLRP3 inflammasome. *Front Microbiol.* (2019) 10:50. 10.3389/fmicb.2019.00050 30761102 PMC6361828

[27] Huang S Zhu B Cheon I Goplen NP Jiang L Zhang R PPAR-γ in macrophages limits pulmonary inflammation and promotes host recovery following respiratory viral infection. *J Virol.* (2019) 93:e00030-19. 10.1128/JVI.00030-19 30787149 PMC6475778

[28] Singh N Jabeen T Sharma S Somvanshi RK Dey S Srinivasan A Specific binding of non-steroidal anti-inflammatory drugs (NSAIDs) to phospholipase A2: structure of the complex formed between phospholipase A2 and diclofenac at 2.7 A resolution. *Acta Crystallogr D Biol Crystallogr.* (2006) 62(Pt 4):410–6. 10.2210/pdb2b17/pdb 16552142

[29] Mäkelä A Kuusi T Schröder T. Inhibition of serum phospholipase-A2 in acute pancreatitis by pharmacological agents in vitro. *Scand J Clin Lab Invest.* (1997) 57:401–7. 10.3109/00365519709084587 9279965

[30] Vijay R Hua X Meyerholz D Miki Y Yamamoto K Gelb M Critical role of phospholipase group IID in age-related susceptibility to severe acute respiratory syndrome – CoV infection. *J.Exp.Med.* (2015) 212:1851–68. 10.1084/jem.20150632 26392224 PMC4612096

